# Empirical Squared Hellinger Distance Estimator and Generalizations to a Family of *α*-Divergence Estimators

**DOI:** 10.3390/e25040612

**Published:** 2023-04-04

**Authors:** Rui Ding, Andrew Mullhaupt

**Affiliations:** Applied Mathematics and Statistics Department, Stony Brook University, Stony Brook, NY 11794, USA; andrew.mullhaupt@stonybrook.edu

**Keywords:** continuous distribution, divergence estimation, Hellinger distance, alpha divergence, information distance, Neyman–Pearson region

## Abstract

We present an empirical estimator for the squared Hellinger distance between two continuous distributions, which almost surely converges. We show that the divergence estimation problem can be solved directly using the empirical CDF and does not need the intermediate step of estimating the densities. We illustrate the proposed estimator on several one-dimensional probability distributions. Finally, we extend the estimator to a family of estimators for the family of α-divergences, which almost surely converge as well, and discuss the uniqueness of this result. We demonstrate applications of the proposed Hellinger affinity estimators to approximately bounding the Neyman–Pearson regions.

## 1. Introduction

We present an empirical estimator for the squared Hellinger distance between two continuous distributions. The work is a direct extension of Perez-Cruz [1] where they provided an empirical KL divergence estimator. Their work is built upon previous works on divergence estimators such as [2,3,4,5,6]. Similar to their estimator, given two samples from two distributions, our estimator does not need to estimate the probability density functions explicitly before estimating the squared Hellinger distance between the two distributions, which makes it simple and fast. We show that the estimator converges to the true squared Hellinger distance almost surely as the sample size increases. We then extend our estimator to the family of α-divergences, to which the squared Hellinger distance belongs. For each of the estimators, we can obtain a reverse estimator using the other direction of the two data samples, and we can also obtain a symmetric estimator by averaging the two one-sided estimators. We present several numerical examples to show the convergence of our estimators. Our newly proposed estimators can be used efficiently to approximate the adjacency of two data samples, leading to various applications in many fields of research.

## 2. Preliminaries on Divergences between Probability Distributions

Recall that the definition of squared Hellinger distance [7] is (for univariate continuous distributions):

$$H^{2}\left(P,Q\right)=\frac{1}{2}\int_{x}{\left(\sqrt{p(x)}-\sqrt{q(x)}\right)}^{2}dx.$$

It is symmetric and always bounded between 0 and 1.

Additionally, recall the definition of Kullback–Leibler divergence [8] is (for univariate continuous distributions):

$$D_{KL}\left(P||Q\right)=\int_{x}p\left(x\right)\log\frac{p(x)}{q(x)}dx.$$

KL divergence and the squared Hellinger distance both belong to a family of f-divergences, which are central to information theory and statistics. Compared with KL divergence, the squared Hellinger distance is symmetric, and Hellinger distance forms a bounded metric between 0 and 1 on the space of probability distributions. Hellinger distance is related to total variation distance as:

$$H^{2}\left(P,Q\right)\leq TVD\left(P,Q\right)\leq \sqrt{2}H\left(P,Q\right),$$

where total variation distance (TVD) is defined as:

$$TVD\left(P,Q\right)=\frac{1}{2}\int_{x}\left|p\left(x\right)-q\left(x\right)\right|dx.$$

The squared Hellinger distance is also closely related to KL divergence and can be bounded by:

$$2H^{2}\left(P,Q\right)\leq D_{KL}\left(P||Q\right).$$

It is also a known result that KL divergence is stronger than Hellinger distance in the sense that convergence in KL divergence implies convergence in Hellinger distance, which further implies convergence in total variation distances. Therefore, Hellinger distance represents a middle ground between KL divergence and total variation distance; it is weaker than KL divergence but stronger than total variation distance in terms of convergence. As shown before, Hellinger distance has close connections to the total variation distance, which is exactly what inference depends on (KL divergence does not admit a useful lower bound on the TVD). It has another attractive property compared with KL divergence, which is the fact that the squared Hellinger distance is always bounded between zero and one for probability distributions that may or may not have the same support, whereas the KL divergence becomes infinite for probability distributions of different supports. In fact, KL divergence can be unbounded for probability distributions supported on the real line. For example, consider *P* to be the standard Cauchy distribution and *Q* to be the standard normal distribution, then $D_{KL}\left(P||Q\right)$ diverges to infinity. Hence, an empirical estimator for KL divergence does not provide meaningful estimates in such a case, while the squared Hellinger distance is always bounded. Due to these desirable properties, we focus mainly on the squared Hellinger distance in this work. The squared Hellinger distance is a member of the family of α-divergences (up to a scaling factor), which are defined in Cichocki and Amari [9] for α∈(0,1) as,

$$D_{A}^{\alpha }\left(P||Q\right)=\frac{1}{\alpha }+\frac{1}{1-\alpha }-\frac{1}{\alpha (1-\alpha )}\int_{x}{\left(\frac{q(x)}{p(x)}\right)}^{1-\alpha }p\left(x\right)dx.$$

The α-divergence can also be related to TVD through the following inequalities, similar to squared Hellinger distance up to a scaling factor (see for example [10,11,12]),

$$\alpha \left(1-\alpha \right)D_{A}^{\alpha }\left(P||Q\right)\leq TVD\left(P,Q\right)\leq \sqrt{\frac{D_{A}^{\alpha }\left(P||Q\right)}{2}}.$$

## 3. Review of Empirical Sample-Based Kullback–Leibler Divergence Estimator of Continuous Distributions

Let $X={\left\{x_{i}\right\}}_{i=1}^{n},X^{\prime }={\left\{x_{j}^{\prime }\right\}}_{j=1}^{m}$ be iid samples from *P* and *Q* in increasing order. Recall that the definition of the empirical CDFs of *P* and *Q* are, respectively,

$$P_{e}\left(x\right)=\frac{1}{n}\sum_{i=1}^{n}U\left(x-x_{i}\right);Q_{e}\left(x\right)=\frac{1}{m}\sum_{j=1}^{m}U\left(x-x_{j}^{\prime }\right),$$

where U(x) is a unit-step function with U(0)=0.5. The continuous piece-wise linear interpolation of the empirical CDF of *P* is denoted as $P_{c}\left(x\right)$. It is zero for any point smaller than a joint lower bound $x_{0}<inf\left\{X,X^{\prime }\right\}$ of the data samples from P,Q, and is one for anything greater than or equal to a joint upper bound $x_{n+1}>sup\left\{X,X^{\prime }\right\}$ of the data samples from P,Q; everywhere in the middle, it is defined as:

$$P_{c}\left(x\right)=a_{i}x+b_{i},x_{i-1}<x<x_{i},$$

where coefficients $a_{i},b_{i}$ are set so that $P_{c}\left(x\right)$ matches the values of $P_{e}\left(x\right)$ at the sampled values $x_{i},i=1,\ldots ,n$. Similarly, we can define the interpolated empirical CDF for *Q*, denoted as $Q_{c}\left(x\right)$. These empirical CDFs converge uniformly and are independent of the distribution of their CDFs.

**Figure 1:**
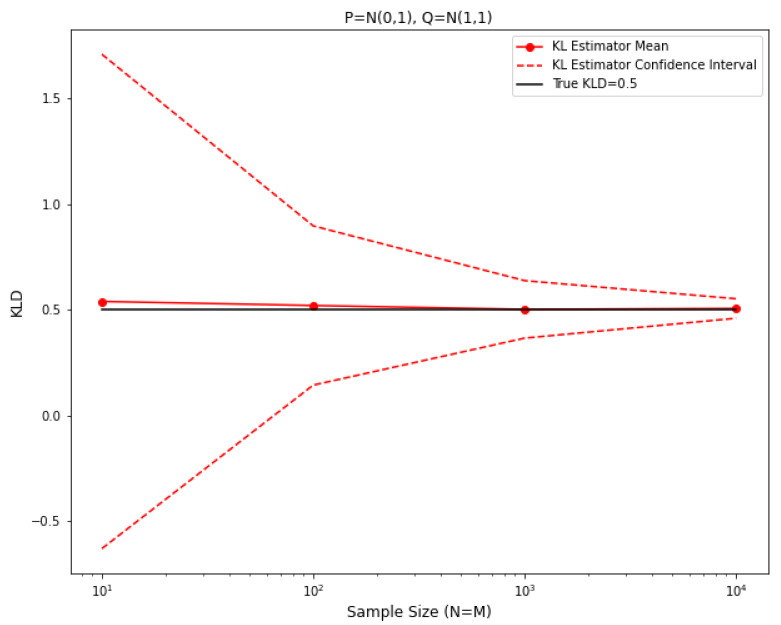
Empirical KLD estimator for two normal distributions.

Perez-Cruz [1] proposed an empirical KL estimator:

$$\hat{D}\left(P||Q\right)=\frac{1}{n}\sum_{i=1}^{n}\log\frac{\delta P_{c}\left(x_{i}\right)}{\delta Q_{c}\left(x_{i}\right)},$$

where $\delta P_{c}\left(x_{i}\right)=\left(P_{c}\left(x_{i}\right)-P_{c}\left(x_{i}-\epsilon \right)\right)/\epsilon$ for any $\epsilon <\min_{i}\left\{x_{i}-x_{i-1}\right\}$ denotes the left slope of $P_{c}$ at $x_{i}$ and $\delta Q_{c}\left(x_{i}\right)$ denotes the left slope of $Q_{c}$ at $x_{i}$. Here, n=|X| and $x_{i}$ are the samples from the *P* distribution. Ref. [1] showed that $\hat{D}\left(P||Q\right)-1\to D\left(P||Q\right)$, almost surely. For this 1-D data setting, an experiment showing the convergence of their estimator is shown in Figure 1 where we plotted estimated values against increasing sample sizes, where P,Q are taken to be normal distributions N(0,1) and N(1,1) respectively.

**Figure 2:**
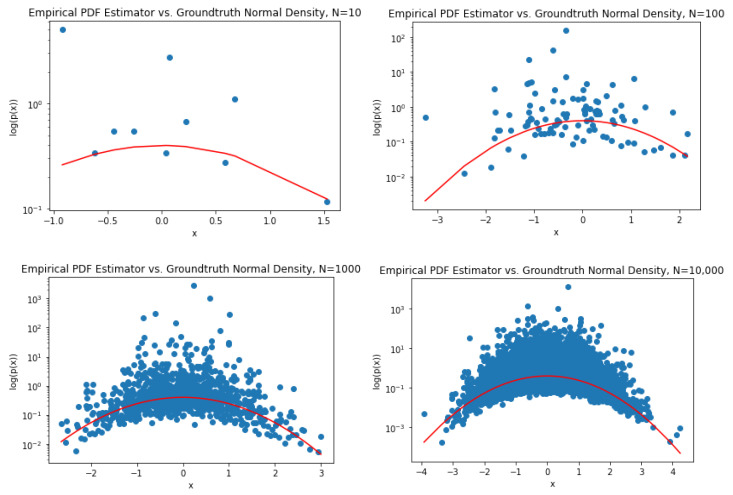
Failure of empirical PDF estimator.

It is worth mentioning that the major innovation and strength of these types of empirical estimators is the fact that there are no convergent density estimators required in the process of estimating the desired divergences. In fact, only the empirical CDF is used and the density model being used in the estimator is completely based on the slopes of the piecewise linear interpolation of the empirical CDF. This empirical density model is far from being convergent as we can see from the following figures in Figure 2, which shows the calculated slopes (in blue) for N=10,100,1000,10,000 data samples from a normal distribution against the ground-truth normal densities (in red), plotted in log scale. Clearly, the empirical density model does not converge to the true densities.

Perez-Cruz [1] also provided an empirical KL estimator for multivariate distribution samples. The estimator is based on a nearest-neighbor approach. For each sample $x_{i}$ in X, where the dimension of the sample is d, let:

$${\hat{p}}_{k}\left(x_{i}\right)=\frac{k}{n-1}\frac{\Gamma (d/2+1)}{\pi^{d/2}r_{k}{\left(x_{i}\right)}^{d}},{\hat{q}}_{k}\left(x_{i}\right)=\frac{k}{m}\frac{\Gamma (d/2+1)}{\pi^{d/2}s_{k}{\left(x_{i}\right)}^{d}},$$

where $r_{k}\left(x_{i}\right),s_{k}\left(x_{i}\right)$ are, respectively, the Euclidean distance to the k-th nearest neighbor of $x_{i}$ in $X\backslash x_{i}$ and $X^{\prime }$, and $\frac{\pi^{d/2}}{\Gamma (d/2+1)}$ is the volume of the unit ball in $R^{d}$. Ref. [1] continued to show that the random variable $\frac{p(x)}{{\hat{p}}_{k}\left(x\right)}$ converges to an independent Gamma(k,k) random variable which has mean 1 and variance $\frac{1}{k}$ for each selected k=1,2,3,…, where *x* is sampled from *P*. Therefore, they proposed the following estimator:

$${\hat{D}}_{k}\left(P||Q\right)=\frac{1}{n}\sum_{i=1}^{n}\log\frac{{\hat{p}}_{k}\left(x_{i}\right)}{{\hat{q}}_{k}\left(x_{i}\right)}=\frac{d}{n}\sum_{i=1}^{n}\log\frac{r_{k}\left(x_{i}\right)}{s_{k}\left(x_{i}\right)}+\log\frac{m}{n-1}.$$

It was shown that, since $\frac{1}{n}\sum_{i=1}^{n}\log\frac{p(x_{i})}{{\hat{p}}_{k}\left(x_{i}\right)}$ and, consequently, $\frac{1}{n}\sum_{i=1}^{n}\log\frac{q(x_{i})}{{\hat{q}}_{k}\left(x_{i}\right)}$ converges to:

$$\frac{1}{(k-1)!}\int_{0}^{\infty }{\left(kx\right)}^{k-1}\log xe^{-kx}kdx=\frac{1}{(k-1)!}\int_{0}^{\infty }z^{k-1}\log ze^{-z}dz-\log k,$$

then ${\hat{D}}_{k}\left(P||Q\right)\to D\left(P||Q\right)$ almost surely.

## 4. Empirical Squared Hellinger Distance Estimator of Continuous Distributions

### 4.1. Estimator for 1D Data

Following Perez-Cruz [1], we have defined a similar estimator for Hellinger affinity using empirical CDFs. Let $X={\left\{x_{i}\right\}}_{i=1}^{n},X^{\prime }={\left\{x_{j}^{\prime }\right\}}_{j=1}^{m}$ be iid samples from *P* and *Q* in increasing order. Recall that the definition of the empirical CDFs of *P* and *Q* are, respectively,

$$P_{e}\left(x\right)=\frac{1}{n}\sum_{i=1}^{n}U\left(x-x_{i}\right),$$

where U(x) is a unit-step function with U(0)=0.5. The continuous piece-wise linear interpolation of the empirical CDF of *P* (and *Q*) is denoted as $P_{c}\left(x\right)$ (and $Q_{c}\left(x\right)$, respectively). It is zero for anything smaller than a joint lower bound $x_{0}<inf\left\{X,X^{\prime }\right\}$ of the data samples from P,Q, and is one for anything greater than or equal to a joint upper bound $x_{n+1}>sup\left\{X,X^{\prime }\right\}$ of the data samples from P,Q; everywhere in the middle, it is defined as:

$$P_{c}\left(x\right)=a_{i}x+b_{i},x_{i-1}<x<x_{i},$$

where coefficients $a_{i},b_{i}$ are set so that $P_{c}\left(x\right)$ matches the values of $P_{e}\left(x\right)$ at the sampled values $x_{i},i=1,\ldots ,n$. $Q_{c}\left(x\right)$ is defined similarly. These empirical CDFs converge uniformly and are independent of the distribution of their CDFs.

Our estimator for the squared Hellinger distance is based on estimating the Hellinger affinity, which is directly related to the quantity of interest by:

$$A\left(P,Q\right)=1-H^{2}\left(P,Q\right)=\int_{x}\sqrt{p(x)q(x)}dx.$$

The new estimator for Hellinger affinity is

$$\hat{A}\left(P,Q\right)=\frac{1}{n}\sum_{i=1}^{n}\sqrt{\frac{\delta Q_{c}\left(x_{i}\right)}{\delta P_{c}\left(x_{i}\right)}},$$

where $\delta P_{c}\left(x_{i}\right)=\left(P_{c}\left(x_{i}\right)-P_{c}\left(x_{i}-\epsilon \right)\right)/\epsilon$ for any $\epsilon <\min_{i}\left\{x_{i}-x_{i-1}\right\}$ denotes the left slope of $P_{c}$ at $x_{i}$ and, similarly, $\delta Q_{c}\left(x_{i}\right)$ denotes the left slope of $Q_{c}$ at $x_{i}$.

We next claim and prove that $\hat{A}$ converges to a scalar multiple of the true Hellinger affinity $\hat{A}\to \frac{\pi }{4}A$. To justify the use of this bias correction constant we need to prove that it results from terms we get from rewriting the estimator:

$$\hat{A}\left(P,Q\right)=\frac{1}{n}\sum_{i=1}^{n}\sqrt{\frac{\Delta Q_{c}\left(x_{mi}^{\prime }\right)/\Delta x_{mi}^{\prime }}{\Delta P_{c}\left(x_{i}\right)/\Delta x_{i}}}$$

$$=\frac{1}{n}\sum_{i=1}^{n}\sqrt{\frac{\Delta Q\left(x_{mi}^{\prime }\right)/\Delta x_{mi}^{\prime }}{\Delta P\left(x_{i}\right)/\Delta x_{i}}}\sqrt{\frac{\Delta Q_{c}\left(x_{mi}^{\prime }\right)}{\Delta Q(x_{mi}^{\prime })}}\sqrt{\frac{\Delta P(x_{i})}{\Delta P_{c}\left(x_{i}\right)}}=\frac{1}{n}\sum_{i=1}^{n}\sqrt{\frac{\Delta Q\left(x_{mi}^{\prime }\right)/\Delta x_{mi}^{\prime }}{\Delta P\left(x_{i}\right)/\Delta x_{i}}}\frac{\sqrt{n\Delta P(x_{i})}}{\sqrt{m\Delta Q(x_{mi}^{\prime })}},$$

where $\Delta x_{i}=x_{i}-x_{i-1},$ $\Delta P_{c}\left(x_{i}\right)=P_{c}\left(x_{i}\right)-P_{c}\left(x_{i-1}\right),$ $\Delta P\left(x_{i}\right)=P\left(x_{i}\right)-P\left(x_{i-1}\right),$ $\Delta x_{mi}^{\prime }=\min\left\{x_{j}^{\prime }|x_{j}^{\prime }\geq x_{i}\right\}-\max\left\{x_{j}^{\prime }|x_{j}^{\prime }<x_{i}\right\},$ $\Delta Q_{c}\left(x_{mi}^{\prime }\right)=Q_{c}\left(\min\left\{x_{j}^{\prime }|x_{j}^{\prime }\geq x_{i}\right\}\right)-Q_{c}(\max\left\{x_{j}^{\prime }|x_{j}^{\prime }<x_{i}\}\right)$ and $\Delta Q\left(x_{mi}^{\prime }\right)=Q\left(\min\left\{x_{j}^{\prime }|x_{j}^{\prime }\geq x_{i}\right\}\right)-Q(\max\left\{x_{j}^{\prime }|x_{j}^{\prime }<x_{i}\}\right)$.

Notice that the first (square root) term in the sum converges almost surely to $\sqrt{\frac{q(x_{i})}{p(x_{i})}}$. We need to show that the above empirical sum converges almost surely to $C\int_{x}\sqrt{p(x)q(x)}dx$, where the constant $C=\frac{\pi }{4}$ is derived from the second term, using similar arguments as Perez-Cruz [1] through waiting time distributions between two consecutive samples from a uniform distribution between 0 and 1.

We outline the proof for the constant term below. Similar to Perez-Cruz [1], we know that, given ${\left\{x_{i}\right\}}_{i=1}^{n}∼P$, $n\Delta P\left(x_{i}\right)∼Exp\left(1\right)$ and is independent of *P* (similarly for Q). With this argument, the last expression for $\hat{A}\left(P,Q\right)$ can be rewritten as (where $z_{i}=n\Delta P\left(x_{i}\right)$)

$$\hat{A}\left(P,Q\right)=\frac{1}{n}\sum_{i=1}^{n}\sqrt{\frac{\Delta Q\left(x_{mi}^{\prime }\right)/\Delta x_{mi}^{\prime }}{\Delta P\left(x_{i}\right)/\Delta x_{i}}}\frac{\sqrt{z_{i}}}{\sqrt{m\Delta Q(x_{mi}^{\prime })}}$$

$$\overset{a.s.}{\to }\left(\frac{1}{n}\sum_{i=1}^{n}\sqrt{\frac{\Delta Q\left(x_{mi}^{\prime }\right)/\Delta x_{mi}^{\prime }}{\Delta P\left(x_{i}\right)/\Delta x_{i}}}\sqrt{z_{i}}\right)*\left(\frac{1}{n}\sum_{i=1}^{n}\frac{1}{\sqrt{m\Delta Q(x_{mi}^{\prime })}}\right).$$

The first sum converges almost surely to:

$$\frac{1}{n}\sum_{i=1}^{n}\sqrt{\frac{\Delta Q\left(x_{mi}^{\prime }\right)/\Delta x_{mi}^{\prime }}{\Delta P\left(x_{i}\right)/\Delta x_{i}}}\sqrt{z_{i}}\overset{a.s.}{\to }\int_{x}\int_{z=0}^{\infty }\sqrt{\frac{q(x)}{p(x)}}\sqrt{z}e^{-z}p\left(x\right)dzdx=\frac{\sqrt{\pi }}{2}A\left(P,Q\right).$$

The second sum can be rewritten as:

$$\frac{1}{n}\sum_{i=1}^{n}\frac{1}{\sqrt{m\Delta Q(x_{mi}^{\prime })}}=\frac{1}{n}\sum_{j=1}^{m}\frac{n\Delta P_{e}\left(x_{j}^{\prime }\right)}{\sqrt{m\Delta Q(x_{j}^{\prime })}}=\frac{1}{m}\sum_{j=1}^{m}\frac{\Delta P_{e}\left(x_{j}^{\prime }\right)/\Delta x_{j}^{\prime }}{\Delta Q\left(x_{j}^{\prime }\right)/\Delta x_{j}^{\prime }}\frac{m\Delta Q(x_{j}^{\prime })}{\sqrt{m\Delta Q(x_{j}^{\prime })}}.$$

The last expression converges almost surely to:

$$\frac{1}{m}\sum_{j=1}^{m}\frac{\Delta P_{e}\left(x_{j}^{\prime }\right)/\Delta x_{j}^{\prime }}{\Delta Q\left(x_{j}^{\prime }\right)/\Delta x_{j}^{\prime }}\frac{m\Delta Q(x_{j}^{\prime })}{\sqrt{m\Delta Q(x_{j}^{\prime })}}\overset{a.s.}{\to }\int_{x}\int_{z=0}^{\infty }\frac{p_{e}\left(x\right)}{q(x)}\sqrt{z}e^{-z}q\left(x\right)dzdx=\frac{\sqrt{\pi }}{2}\int_{x}p_{e}\left(x\right)dx=\frac{\sqrt{\pi }}{2}.$$

Notice here that $p_{e}\left(x\right)$ is a density model but does not need to converge to p(x) for the above expression to converge to the desired constant.

Combining all previous results, we have shown that $\hat{A}\left(P,Q\right)$ converges almost surely to:

$$\hat{A}\left(P,Q\right)\overset{a.s.}{\to }\frac{\sqrt{\pi }}{2}\frac{\sqrt{\pi }}{2}A\left(P,Q\right)=\frac{\pi }{4}A\left(P,Q\right).$$

Hence, we obtained the desired constant $C=\frac{\pi }{4}\approx 0.785$. The final estimator for squared Hellinger distance is ${\hat{H}}^{2}\left(P,Q\right)=1-\frac{4\hat{A}\left(P,Q\right)}{\pi }$.

Notice that Hellinger distance is a symmetric distance metric for any distributions *P* and Q, hence the estimator above is only one side of the story. Following exactly the same arguments, we can show that the opposite direction estimator,

$$\hat{A}\left(Q,P\right)=\frac{1}{m}\sum_{j=1}^{m}\sqrt{\frac{\delta P_{c}\left(x_{j}^{\prime }\right)}{\delta Q_{c}\left(x_{j}^{\prime }\right)}},$$

also converges almost surely to $\frac{\pi }{4}A\left(Q,P\right)$, and since A(P,Q)=A(Q,P) we can obtain a symmetric estimator of Hellinger affinity that converges almost surely to $\frac{\pi }{4}A\left(Q,P\right)$:

$${\hat{A}}_{S}\left(P,Q\right)=\frac{\hat{A}\left(P,Q\right)+\hat{A}\left(Q,P\right)}{2}.$$

Therefore, we can construct a corresponding estimator for the squared Hellinger distance as:

$${\hat{H}}_{S}^{2}\left(P,Q\right)=1-\frac{4{\hat{A}}_{S}\left(P,Q\right)}{\pi },$$

which enjoys all of the properties shown above for the two estimators separately. Since the symmetric version uses more information from the two samples, it is supposed to be able to provide better estimates than the two single-sided estimators in terms of the rate of convergence.

### 4.2. Numerical Experiments

We show asymptotic convergence of the new estimator ${\hat{H}}^{2}\left(P,Q\right)=1-\frac{4\hat{A}\left(P,Q\right)}{\pi }$, and its symmetric version, to the true $H^{2}$ value as the data sample size grows in the below experiments. In each of the experiments, we took two distributions of the same family and compared the estimated squared Hellinger distance value against the ground truth value. We plotted mean estimated values for sample size N=M=10,32,100,316,1000,3162,10,000 (x-axis) used for each pair of distributions over 100 instances, and we also plotted the 95% confidence interval of the estimates. For each experiment, the squared Hellinger distance estimators ${\hat{H}}^{2}\left(P,Q\right),{\hat{H}}^{2}\left(Q,P\right)$ are plotted in red and blue, and the symmetric squared Hellinger distance estimator ${\hat{H}}_{S}^{2}\left(P,Q\right)$ is plotted in purple. We also recall the fact that when P,Q are taken to be normal distributions $N\left(\mu_{1},\sigma_{1}^{2}\right),N\left(\mu_{2},\sigma_{2}^{2}\right)$, the squared Hellinger distance has an analytic form:

$$H^{2}\left(P,Q\right)=1-\sqrt{\frac{2\sigma_{1}\sigma_{2}}{\sigma_{1}^{2}+\sigma_{2}^{2}}}e^{-\frac{1}{4}\frac{{\left(\mu_{1}-\mu_{2}\right)}^{2}}{\sigma_{1}^{2}+\sigma_{2}^{2}}}.$$

**Figure 3:**
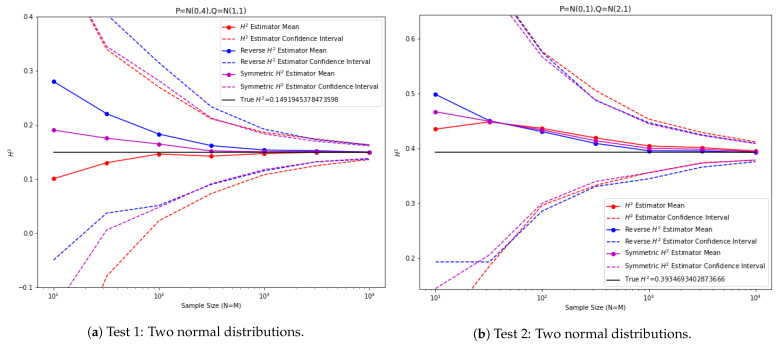
Empirical squared Hellinger estimator tests between 1D normal distributions.

In the first experiment (Figure 3), P,Q are taken to be normal distributions N(0,4) and N(1,1), respectively. In the second experiment P,Q are taken to be normal distributions N(0,1) and N(2,1), respectively.

**Figure 4:**
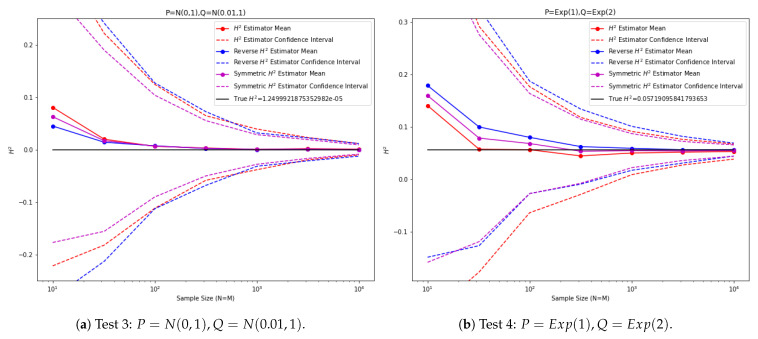
Empirical squared Hellinger estimator tests between 1D distributions.

In the third experiment (Figure 4), P,Q are taken to be normal distributions N(0,1) and N(0.01,1), respectively. In the fourth experiment, P,Q are taken to be exponential distributions Exp(1) and Exp(2), respectively.

**Figure 5:**
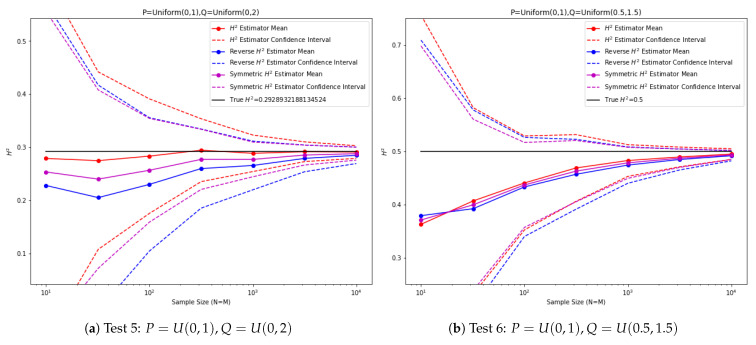
Empirical squared Hellinger estimator tests between 1D distributions.

In the fifth experiment (Figure 5), P,Q are taken to be uniform distributions U(0,1) and U(0,2), respectively. In the sixth experiment, P,Q are taken to be uniform distributions U(0,1) and U(0.5,1.5), respectively. Notice that the squared Hellinger distance is well-defined for distributions of different support.

**Figure 6:**
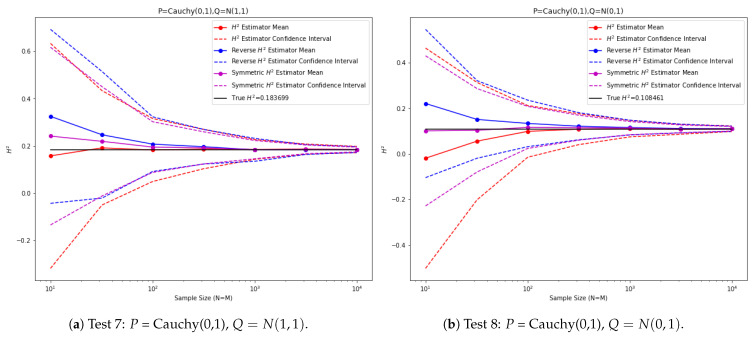
Empirical squared Hellinger estimator tests between 1D Cauchy and normal distributions.

In the last two experiments (Figure 6), we considered two distributions from different distribution families. Here, P=Cauchy(0,1) is the standard Cauchy distribution. In the seventh experiment, Q=N(1,1) and in the last experiment Q=N(0,1). The true squared Hellinger distances are computed using numerical integration.

We can observe from the previous experiments that, depending on the distributions, either the estimator ${\hat{H}}^{2}\left(P,Q\right)$ or the reverse direction estimator ${\hat{H}}^{2}\left(Q,P\right)$ can turn out to be better, which is a consequence of our choice to take the left slope of the empirical CDF so the relative location of the two distributions will determine which estimator is more accurate. The symmetric squared Hellinger estimator provides a middle ground between the two one-sided estimators and it also exhibits smaller variances.

As mentioned before, the proposed estimator does not use the information of the underlying distribution and does not need to estimate the density first before estimating the squared Hellinger distance. As a comparison with an estimator that knows the distribution, we performed experiments with Gaussian distributions where we could use the sample mean and sample variance to estimate the distributions and then compute the squared Hellinger distance analytically using the estimated parameters. The estimator is constructed as follows,

$${\hat{H}}_{naive}^{2}\left(P,Q\right)=1-\sqrt{\frac{2{\hat{\sigma }}_{1}\hat{\sigma_{2}}}{{\hat{\sigma }}_{1}^{2}+{\hat{\sigma }}_{2}^{2}}}e^{-\frac{1}{4}\frac{{\left({\hat{\mu }}_{1}-{\hat{\mu }}_{2}\right)}^{2}}{{\hat{\sigma }}_{1}^{2}+{\hat{\sigma }}_{2}^{2}}},$$

where ${\hat{\mu }}_{1},{\hat{\sigma }}_{1},{\hat{\mu }}_{2},{\hat{\sigma }}_{2}$ are sample estimates of mean and standard deviation from the two datasets. This estimator knows extra information about the data coming from Gaussian distributions.

**Figure 7:**
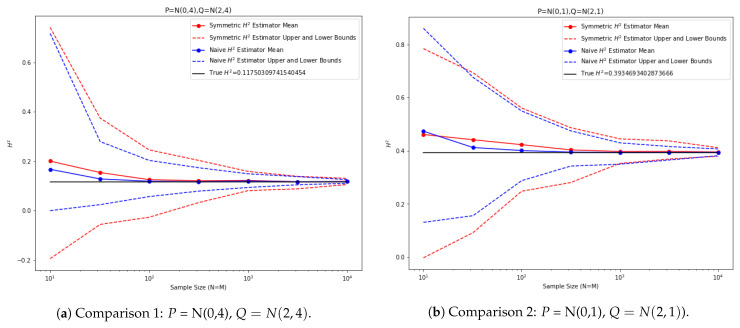
Comparisons between empirical and naive estimators for 1D normal distributions.

However, as we can see from the plots in Figure 7, the proposed squared Hellinger distance estimator performs similarly to the estimator that knows the distribution family. In the first experiment, the two distributions are N(0,4),N(2,4). In the second experiment, the two distributions are N(0,1),N(2,1). For both plots, we plotted the proposed symmetric squared Hellinger distance estimator in red and the naive estimator using sampled parameters in blue. The upper bound and lower bound of each estimator, performed over 100 iterations, are plotted in dashed lines.

**Figure 8:**
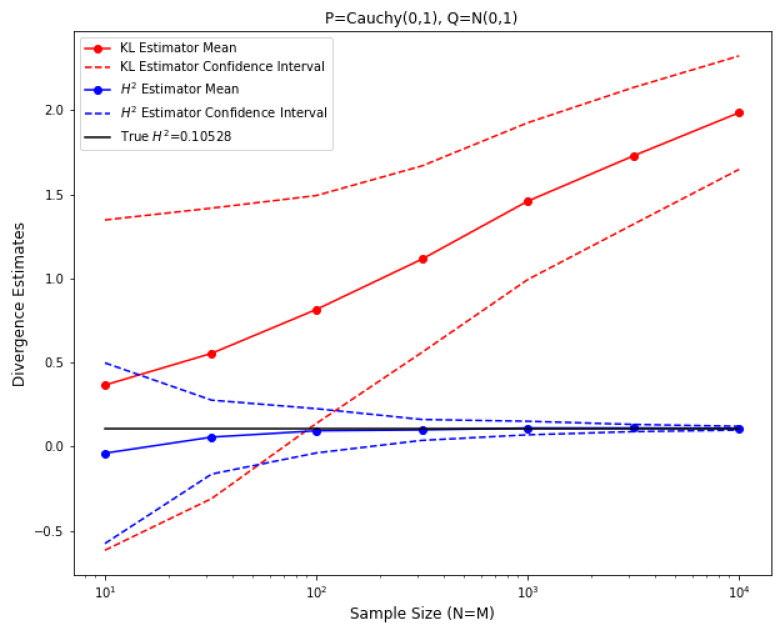
Comparison of empirical $D_{KL}$ estimator against empirical $H^{2}$ estimator, P=Cauchy(0,1),Q=N(0,1).

Finally, we consider the setting in Test 8, where *P* is a standard Cauchy distribution and *Q* is a standard normal distribution, and we compare the behavior of the empirical squared Hellinger estimator ${\hat{H}}^{2}\left(P,Q\right)$ with the empirical KL divergence estimator $\hat{D}\left(P||Q\right)$ as in [1]. As mentioned in the discussion in Section 2, for this case, the KL divergence diverges to infinity while the squared Hellinger distance is bounded. With the same experiment setup, we plotted the resulting divergence estimates and confidence intervals for both KL divergence and squared Hellinger distance in Figure 8, where the ground truth $H^{2}\left(P,Q\right)$ value (approximated by numerical integration) is plotted in black and the ground truth $D_{KL}\left(P||Q\right)$ value is infinity.

As we can observe from Figure 8, while the empirical squared Hellinger estimator converges to the ground truth value quickly, the empirical KL divergence estimator cannot converge to some value due to the fact that the ground truth value is infinity. This justifies the desirability of considering the squared Hellinger distance, which is always bounded.

### 4.3. Estimator for Vectorial Data

Utilizing the results proved for the vectorial data case in [1], we propose the following estimator for squared Hellinger distance in multivariate cases (for a chosen *k*). Similar to the definitions in [1], let the kNN density estimator be defined as:

$${\hat{p}}_{k}\left(x_{i}\right)=\frac{k}{n-1}\frac{\Gamma (d/2+1)}{\pi^{d/2}r_{k}{\left(x_{i}\right)}^{d}},{\hat{q}}_{k}\left(x_{i}\right)=\frac{k}{m}\frac{\Gamma (d/2+1)}{\pi^{d/2}s_{k}{\left(x_{i}\right)}^{d}},$$

where $r_{k}\left(x_{i}\right),s_{k}\left(x_{i}\right)$ are, respectively, the Euclidean distance to the k-th nearest neighbor of $x_{i}$ in $X\backslash x_{i}$ and $X^{\prime }$. Let:

$${\hat{A}}_{k}\left(P,Q\right)=\frac{1}{n}\sum_{i=1}^{n}\sqrt{\frac{{\hat{q}}_{k}\left(x_{i}\right)}{{\hat{p}}_{k}\left(x_{i}\right)}}=\frac{1}{n}\sum_{i=1}^{n}\sqrt{\frac{\left(n-1\right)r_{k}{\left(x_{i}\right)}^{d}}{ms_{k}{\left(x_{i}\right)}^{d}}}$$

$${\hat{A}}_{k}\left(P,Q\right)=\frac{1}{n}\sum_{i=1}^{n}\sqrt{\frac{q(x_{i})}{p(x_{i})}}\sqrt{\frac{{\hat{q}}_{k}\left(x_{i}\right)}{q(x_{i})}}\sqrt{\frac{p(x_{i})}{{\hat{p}}_{k}\left(x_{i}\right)}};$$

since $\frac{p(x)}{{\hat{p}}_{k}\left(x\right)},\frac{q(x)}{{\hat{q}}_{k}\left(x\right)}$ are independent Gamma(k,k) random variables that are also independent from P,Q, we conclude that ${\hat{A}}_{k}\left(P,Q\right)$ converges almost surely to:

$${\hat{A}}_{k}\left(P,Q\right)\to A\left(P,Q\right)\sqrt{k}\int_{0}^{\infty }z^{-1/2}\frac{z^{k-1}e^{-z}}{(k-1)!}dz\frac{1}{\sqrt{k}}\int_{0}^{\infty }z^{1/2}\frac{z^{k-1}e^{-z}}{(k-1)!}dz$$

$$=\frac{\Gamma (k-\frac{1}{2})}{(k-1)!}\frac{\Gamma (k+\frac{1}{2})}{(k-1)!}A\left(P,Q\right).$$

So, ${\hat{A}}_{k}\left(P,Q\right)$ converges almost surely to the true Hellinger affinity up to a constant multiplier, similar to the 1D case. Therefore, we propose the following estimator for squared Hellinger distance, which converges almost surely to the true squared Hellinger distance:

$${\hat{H}}_{k}^{2}\left(P,Q\right)=1-\frac{{\hat{A}}_{k}\left(P,Q\right)\left(k-1\right)!\left(k-1\right)!}{\Gamma \left(k-\frac{1}{2}\right)\Gamma \left(k+\frac{1}{2}\right)}\to H^{2}\left(P,Q\right).$$

Similar to the 1D case, we can extend this estimator to a symmetric version that also shares the desired convergence properties:

$${\hat{H}}_{k,S}^{2}\left(P,Q\right)=\frac{{\hat{H}}_{k}^{2}\left(P,Q\right)+{\hat{H}}_{k}^{2}\left(Q,P\right)}{2}.$$

### 4.4. Numerical Experiments for Vectorial Data

Similar to the experiment setting in Section 4.2, we show the convergence of the proposed estimators in Section 4.3. Sample size N=M=10,32,100,316,1000,3162 (plotted on the x-axis) is used for each pair of distributions. The analytical formula for the squared Hellinger distance for two multivariate Gaussians $N\left(\mu_{1},\Sigma_{1}\right),N\left(\mu_{2},\Sigma_{2}\right)$ is:

$$H^{2}\left(P,Q\right)=1-\frac{|\Sigma_{1}{|}^{1/4}{\left|\Sigma_{2}\right|}^{1/4}}{|\frac{1}{2}\Sigma_{1}+\frac{1}{2}\Sigma_{2}{|}^{1/2}}e^{-\frac{1}{8}{\left(\mu_{1}-\mu_{2}\right)}^{T}{\left(\frac{1}{2}\Sigma_{1}+\frac{1}{2}\Sigma_{2}\right)}^{-1}\left(\mu_{1}-\mu_{2}\right)}.$$

**Figure 9:**
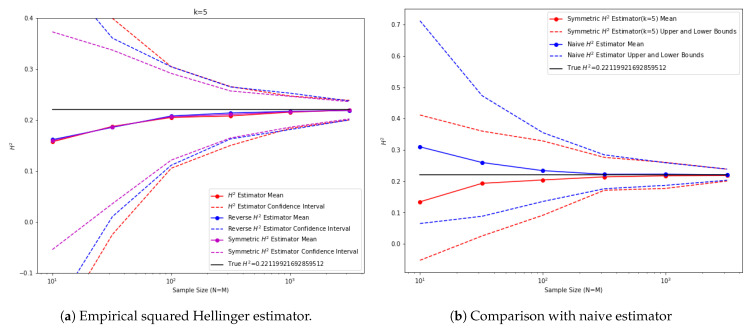
Vectorial squared Hellinger estimator (k=5) tests on 2D normal distributions.

In the experiment in Figure 9, we picked 2D normal distributions *P* and *Q* with $\mu_{1}={\left(0,0\right)}^{T},\mu_{2}={\left(1,1\right)}^{T},\Sigma_{1}=\Sigma_{2}=I_{2}$. For the proposed k-nearest neighbor estimator, we picked k=5. The performance of the proposed estimators and a comparison with the naive estimator are plotted below. The naive estimator estimates the mean and covariance based on the data samples and estimates the squared Hellinger distance based on the analytic formula:

$${\hat{H}}_{naive}^{2}\left(P,Q\right)=1-\frac{|{\hat{\Sigma }}_{1}{|}^{1/4}{\left|{\hat{\Sigma }}_{2}\right|}^{1/4}}{|\frac{1}{2}{\hat{\Sigma }}_{1}+\frac{1}{2}{\hat{\Sigma }}_{2}{|}^{1/2}}e^{-\frac{1}{8}{\left({\hat{\mu }}_{1}-{\hat{\mu }}_{2}\right)}^{T}{\left(\frac{1}{2}{\hat{\Sigma }}_{1}+\frac{1}{2}{\hat{\Sigma }}_{2}\right)}^{-1}\left({\hat{\mu }}_{1}-{\hat{\mu }}_{2}\right)}.$$

From these results we can observe that, similar to the 1D cases, the symmetric estimator seems to perform the best and is comparable to the naive estimator in terms of convergence.

**Figure 10:**
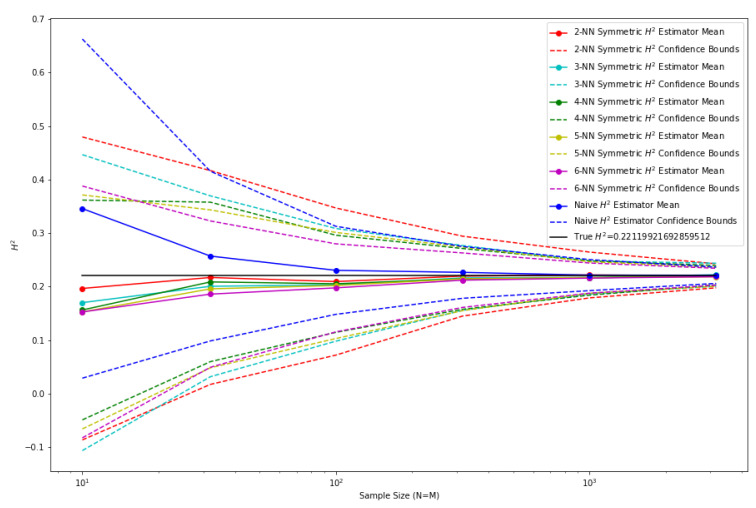
Comparison of kNN-based squared Hellinger distance estimators.

In general, a larger *k* leads to a smaller variance in the proposed estimator for multivariate data. To balance the convergence rate with computational cost, we can select *k* to be around 4 to 6 which converges faster than a smaller *k* and is also easy to compute. This behavior is shown in Figure 10, where we compared the performance of the proposed estimator using k=2,3,4,5,6 for the same experiment setting as above.

**Figure 11:**
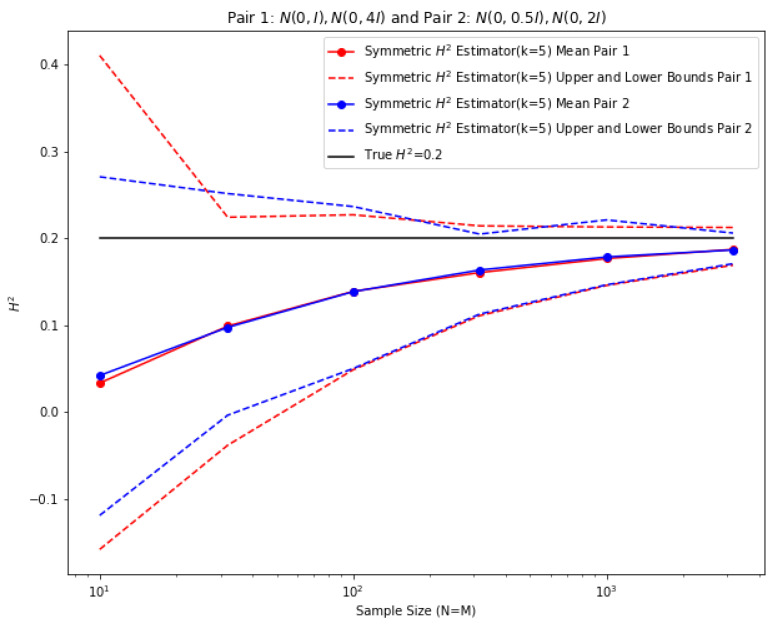
Two pairs of concentric Gaussians with invariant squared Hellinger distance.

Another test we conducted was to check if the squared Hellinger distance estimate behavior in a non-asymptotic sense is similar for two pairs of concentric Gaussians that have the same squared Hellinger distance. For this experiment, we picked the first pair of Gaussians to be N(0,I) and N(0,4I), and the second pair of Gaussians to be $N(0,\frac{1}{2}I)$ and N(0,2I). The squared Hellinger distance between each pair of Gaussians is 0.2 and, since these two pairs correspond to a single coordinate transformation on the sample space, we expect similar behavior of the estimator in terms of convergence on both pairs. The result is shown in Figure 11. As expected, the empirical estimator for vectorial data has very similar convergence behavior for each of the two pairs of Gaussians to the same ground-truth value.

## 5. Empirical α-Divergence Estimator of Continuous Distributions

### 5.1. Estimator for 1D Data

We generalized the results obtained before to a family of α-divergences to which the squared Hellinger distance belongs. Following Cichoki and Amari [9], we define an α-divergence between two probability distributions as:

$$D_{A}^{\alpha }\left(P||Q\right)=\frac{1}{\alpha (\alpha -1)}\int_{x}\left(p^{\alpha }\left(x\right)q^{1-\alpha }\left(x\right)-\alpha p\left(x\right)+\left(\alpha -1\right)q\left(x\right)\right)dx.$$

We want to obtain an empirical estimator similar to that in Section 3 that uses only the empirical CDFs of *P* and *Q* and estimates this quantity directly for any α∈(0,1). Notice that for α=0.5, $D_{A}^{\alpha }\left(P||Q\right)=4H^{2}\left(P,Q\right)$, which corresponds to the squared Hellinger distance.

Notice that we can rewrite the α-divergence above as:

$$D_{A}^{\alpha }\left(P||Q\right)=\frac{1}{\alpha }+\frac{1}{1-\alpha }-\frac{1}{\alpha (1-\alpha )}\int_{x}{\left(\frac{q(x)}{p(x)}\right)}^{1-\alpha }p\left(x\right)dx.$$

Clearly, we are interested in the last quantity, so we only need to have an estimator for that term that converges almost surely.

For this purpose, let us define an estimator:

$${\hat{A}}^{\alpha }\left(P||Q\right)=\frac{1}{n}\sum_{i=1}^{n}{\left(\frac{\delta Q_{c}\left(x_{i}\right)}{\delta P_{c}\left(x_{i}\right)}\right)}^{1-\alpha }.$$

Notice that, in the general cases, the α-divergence is not symmetric.

Following similar procedures as in Section 3, we can rewrite the estimator as:

$${\hat{A}}^{\alpha }\left(P||Q\right)=\frac{1}{n}\sum_{i=1}^{n}{\left(\frac{\Delta Q(x_{mi}^{\prime }/\Delta x_{mi}^{\prime })}{\Delta P\left(x_{i}\right)/\Delta x_{i}}\right)}^{1-\alpha }{\left(\frac{n\Delta P(x_{i})}{m\Delta Q(x_{mi}^{\prime })}\right)}^{1-\alpha }$$

$$=\frac{1}{n}\sum_{i=1}^{n}{\left(\frac{\Delta Q(x_{mi}^{\prime }/\Delta x_{mi}^{\prime })}{\Delta P\left(x_{i}\right)/\Delta x_{i}}\right)}^{1-\alpha }{\left(\frac{z_{i}}{m\Delta Q(x_{mi}^{\prime })}\right)}^{1-\alpha }.$$

This sum converges almost surely to (since the exponential waiting distributions are independent of the data distribution):

$$\left(\frac{1}{n}\sum_{i=1}^{n}{\left(\frac{\Delta Q(x_{mi}^{\prime }/\Delta x_{mi}^{\prime })}{\Delta P\left(x_{i}\right)/\Delta x_{i}}\right)}^{1-\alpha }z_{i}^{1-\alpha }\right)\left(\frac{1}{m}\sum_{j=1}^{m}{\left(\frac{1}{m\Delta Q(x_{j}^{\prime })}\right)}^{1-\alpha }m\Delta Q\left(x_{j}^{\prime }\right)\frac{\Delta P_{e}\left(x_{j}^{\prime }\right)}{\Delta Q(x_{j}^{\prime })}\right).$$

Following the same arguments as in Section 3, we can show that the proposed estimator converges almost surely to:

$${\hat{A}}^{\alpha }\left(P||Q\right)\overset{a.s.}{\to }\int_{x}{\left(\frac{q(x)}{p(x)}\right)}^{1-\alpha }p\left(x\right)dx\int_{z=0}^{\infty }z^{1-\alpha }e^{-z}dz\int_{z=0}^{\infty }z^{\alpha }e^{-z}dz\int_{x}p_{e}\left(x\right)dx$$

$$=C_{1-\alpha }C_{\alpha }\int_{x}{\left(\frac{q(x)}{p(x)}\right)}^{1-\alpha }p\left(x\right)dx,$$

where we define the constants $C_{1-\alpha }=\int_{z=0}^{\infty }z^{1-\alpha }e^{-z}dz=\Gamma \left(2-\alpha \right),C_{\alpha }=\int_{z=0}^{\infty }z^{\alpha }e^{-z}dz=\Gamma \left(1+\alpha \right),\forall \alpha \in \left(-1,2\right)$.

Therefore, we know that the estimator

$${\hat{D}}_{A}^{\alpha }\left(P||Q\right)=\frac{1}{\alpha }+\frac{1}{1-\alpha }-\frac{1}{\alpha (1-\alpha )}\frac{{\hat{A}}^{\alpha }\left(P||Q\right)}{C_{\alpha }C_{1-\alpha }}$$

converges almost surely to the true α-divergence value, $D_{A}^{\alpha }\left(P||Q\right)$.

Although the α-divergence is not symmetric, it has the property that

$$D_{A}^{\alpha }\left(P||Q\right)=D_{A}^{1-\alpha }\left(Q||P\right).$$

So, given the same two sample data sets, we can get another estimator for the same quantity based on ${\hat{D}}_{A}^{1-\alpha }\left(Q||P\right)=\frac{1}{\alpha }+\frac{1}{1-\alpha }-\frac{1}{\alpha (1-\alpha )}\frac{{\hat{A}}^{1-\alpha }\left(Q||P\right)}{C_{\alpha }C_{1-\alpha }}$, where we are estimating based on the sampling distribution from *Q* instead of *P*. Since ${\hat{D}}_{A}^{\alpha }\left(P||Q\right),{\hat{D}}_{A}^{1-\alpha }\left(Q||P\right)$ converges to the same divergence value, we can again create a symmetric estimator based on averaging these two estimators ${\hat{D}}_{A,S}^{\alpha }\left(P||Q\right)=\frac{{\hat{D}}_{A}^{\alpha }\left(P||Q\right)+{\hat{D}}_{A}^{1-\alpha }\left(Q||P\right)}{2}$ and it is expected to perform similarly if not better. Lastly, notice that, when α=0.5, we obtain $C_{\alpha }=C_{1-\alpha }=\frac{\sqrt{\pi }}{2}$ and ${\hat{D}}_{A}^{0.5}(P||Q)=4(1-\frac{4}{\pi }{\hat{A}}^{0.5}\left(P||Q)\right)$, which corresponds to the squared Hellinger estimator we have seen in Section 3, scaled by 4.

### 5.2. Numerical Experiments

We show asymptotic convergence of the new estimator ${\hat{D}}_{A}^{\alpha }\left(P||Q\right)$, and its symmetric version, to the true α-divergence value as the data sample size grows in the below experiments. In each of the below experiments, we took two distributions of the same family and compared the estimated α-divergence value against the ground truth value. Mean estimated values for sample size N=M=10,32,100,316,1000,3162,10,000,31,623 (plotted on the x-axis) used for each pair of distributions over 100 instances and we also plotted the 95% confidence interval of the estimates. For each experiment, the α-divergence estimators ${\hat{D}}_{A}^{\alpha }\left(P||Q\right),{\hat{D}}_{A}^{1-\alpha }\left(Q||P\right)$ are plotted in red and blue, and the symmetric α-divergence estimator ${\hat{D}}_{A,S}^{\alpha }\left(P||Q\right)$ is plotted in purple.

**Figure 12:**
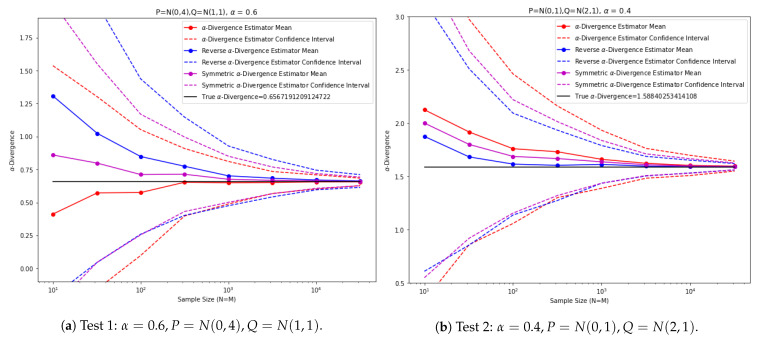
α-divergence estimator tests on 1D normal distributions.

In the first experiment, P,Q are taken to be normal distributions N(0,4) and N(1,1), respectively, and α=0.6. In the second experiment, P,Q are taken to be normal distributions N(0,1) and N(2,1), respectively, and α=0.4. The results are plotted in Figure 12. Notice that for two normal distributions $P∼N\left(\mu_{1},\sigma_{1}^{2}\right),Q∼N\left(\mu_{2},\sigma_{2}^{2}\right)$, we have an analytical formula for the α-divergence:

$$D_{A}^{\alpha }\left(P||Q\right)=\frac{1}{\alpha (1-\alpha )}\left(1-\frac{\sigma_{2}^{\alpha }\sigma_{1}^{1-\alpha }}{\sqrt{\alpha \sigma_{2}^{2}+\left(1-\alpha \right)\sigma_{1}^{2}}}e^{-\frac{\alpha (1-\alpha )}{\alpha \sigma_{2}^{2}+\left(1-\alpha \right)\sigma_{1}^{2}}\frac{{\left(\mu_{1}-\mu_{2}\right)}^{2}}{2}}\right).$$

**Figure 13:**
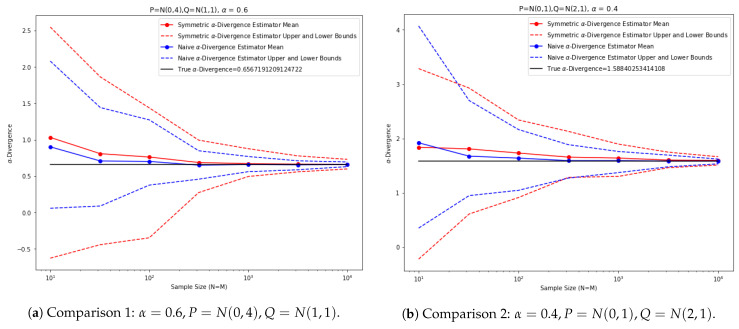
Comparisons between empirical and naive estimators for 1D normal distributions.

Again, we provide a comparison with an estimator that knows the distribution family. We performed experiments with Gaussian distributions where we could use the sample mean and sample variance to estimate the distributions and then compute the α-divergences analytically using the estimated parameters. The estimator is constructed as follows:

$${\hat{D}}_{A,naive}^{\alpha }\left(P||Q\right)=\frac{1}{\alpha (1-\alpha )}\left(1-\frac{{\hat{\sigma }}_{2}^{\alpha }{\hat{\sigma }}_{1}^{1-\alpha }}{\sqrt{\alpha {\hat{\sigma }}_{2}^{2}+\left(1-\alpha \right){\hat{\sigma }}_{1}^{2}}}e^{-\frac{\alpha (1-\alpha )}{\alpha {\hat{\sigma }}_{2}^{2}+\left(1-\alpha \right){\hat{\sigma }}_{1}^{2}}\frac{{\left({\hat{\mu }}_{1}-{\hat{\mu }}_{2}\right)}^{2}}{2}}\right)$$

where ${\hat{\mu }}_{1},{\hat{\sigma }}_{1},{\hat{\mu }}_{2},{\hat{\sigma }}_{2}$ are sample estimates of mean and standard deviation from the two datasets. This estimator knows extra information about the data coming from Gaussian distributions. However, as we can see from the plots in Figure 13, the proposed α-divergence estimator performs similarly to the estimator that knows the distribution family.

In the first experiment, the two distributions are N(0,4),N(1,1) and α=0.6. In the second experiment, the two distributions are N(0,1),N(2,1) and α=0.4. For both plots, we plotted the proposed symmetric α-divergence estimator in red and the naive estimator using sampled parameters in blue. The upper bound and lower bound of each estimator, performed over 100 iterations, are plotted in dashed lines.

### 5.3. Estimator for Vectorial Data

Similarly to Section 4.3, we propose α-divergence estimators for samples from multivariate distributions. For this purpose, let us define:

$${\hat{A}}_{k}^{\alpha }\left(P||Q\right)=\frac{1}{n}\sum_{i=1}^{n}{\left(\frac{{\hat{q}}_{k}\left(x_{i}\right)}{{\hat{p}}_{k}\left(x_{i}\right)}\right)}^{1-\alpha }.$$

Using similar arguments, we can show that this estimator converges almost surely to:

$${\hat{A}}_{k}^{\alpha }\left(P||Q\right)\to \left(k^{1-\alpha }\int_{0}^{\infty }z^{\alpha -1}\frac{z^{k-1}e^{-z}}{(k-1)!}dz\right)\left(k^{\alpha -1}\int_{0}^{\infty }z^{1-\alpha }\frac{z^{k-1}e^{-z}}{(k-1)!}dz\right)\int_{x}{\left(\frac{q(x)}{p(x)}\right)}^{1-\alpha }p\left(x\right)dx$$

$$=\frac{\Gamma (k+\alpha -1)}{(k-1)!}\frac{\Gamma (k-\alpha +1)}{(k-1)!}\int_{x}{\left(\frac{q(x)}{p(x)}\right)}^{1-\alpha }p\left(x\right)dx.$$

Therefore, we propose the following estimator for α-divergences, which converges almost surely:

$${\hat{D}}_{A,k}^{\alpha }\left(P||Q\right)=\frac{1}{\alpha }+\frac{1}{1-\alpha }-\frac{1}{\alpha (1-\alpha )}\frac{{\hat{A}}_{k}^{\alpha }\left(P||Q\right)\left(k-1\right)!\left(k-1\right)!}{\Gamma (k+\alpha -1)\Gamma (k-\alpha +1)}\to D_{A}^{\alpha }\left(P||Q\right).$$

Similarly, we can extend this estimator to a symmetric version, for any fixed *k*:

$${\hat{D}}_{A,k,S}^{\alpha }\left(P||Q\right)=\frac{{\hat{D}}_{A,k}^{\alpha }\left(P||Q\right)+{\hat{D}}_{A,k}^{1-\alpha }\left(Q||P\right)}{2}.$$

As a remark, for the vectorial case, the above kNN density-based empirical estimator for α-divergences (and the squared Hellinger distance in Section 4.3 as a special case) agree with the estimators proposed in [13], although the proof of convergence differs. Nonetheless, the univariate estimators we proposed in Section 4.1 and Section 5.1 are different from trivial reductions of the kNN-based estimators in Section 4.3 and Section 5.3 when taking d=1 and k=1.

## 6. Limitation of the Proposed Methodologies and Uniqueness of the α-Divergences

### 6.1. Failure of a Similar Estimator for Total Variation Distance

As we have shown so far, by using the trick of waiting time distributions, we can bias-correct an empirical mean type estimator to produce an almost-sure convergence estimator for KL divergence, squared Hellinger distance, and in general the α-divergences. However, the same kind of trick does not work for other f-divergences that have an f-function without certain desired properties such as f(ab)=f(a)+f(b) for KL divergence or f(ab)=f(a)f(b) for Hellinger affinity, which we shall discuss in more detail later. As a simple demonstration, consider the Total Variation Distance (TVD), which, for two continuous distributions *P* and Q, is defined as:

$$TVD\left(P,Q\right)=\frac{1}{2}\int_{x}\left|p\left(x\right)-q\left(x\right)\right|dx.$$

Notice that the TVD is always bounded between 0 and 1.

We considered paired distributions in two different families in 1D, namely normal distributions and exponential distributions. For different choices of parameters, we plotted the performance of a biased estimator using the empirical CDFs against the true TVD value. For every parameter setting, we looked at the case where N=M=10,000 and averaged over 100 instances. The estimator is defined as:

$$\hat{TVD}\left(P,Q\right)=\frac{1}{n}\sum_{i=1}^{n}\frac{1}{2}\left|\frac{\delta Q_{c}\left(x_{i}\right)}{\delta P_{c}\left(x_{i}\right)}-1\right|$$

**Figure 14:**
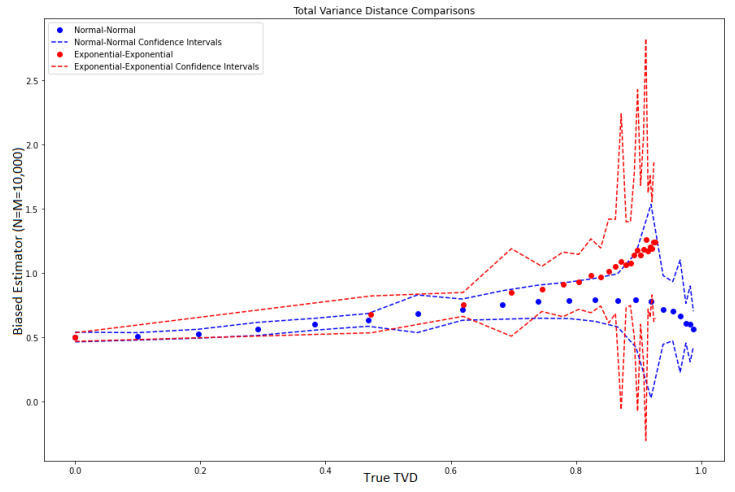
Raw empirical TVD estimator.

Specifically, for the normal distributions, we fixed $\mu_{1}=0,\sigma_{1}=1,\sigma_{2}=1$ and varied $\mu_{2}$ from 0 to 5. For the exponential distributions, we fixed $\lambda_{1}=0.1$ and varied $\lambda_{2}$ from 0.1 to 7. This generated a range of true TVD values that are spaced between 0 and 1 for each distribution family. Figure 14 plots the biased estimator values (on the y-axis) against true TVD values (on the x-axis) for pairs of normal distributions P,Q in blue and pairs of exponential distributions P,Q in red. The confidence intervals are also plotted. We observe that, for the same true TVD values, the biased estimator produced different values for different distribution families, where the relationship looks nonlinear and depends on the distribution family itself. This is an indication that the proposed estimator cannot be uniformly corrected with a simple additive and/or multiplicative constant as we performed for squared Hellinger distance (and in general α-divergences) and [1] for KL divergences. Therefore, we conclude that, so far, the proposed methodologies work for KL divergence, squared Hellinger distance, and in general α-divergences only, but cannot be extended to the general f-divergences in a straightforward way.

### 6.2. Uniqueness of α-Divergences

We provide a more detailed explanation as to why the α-divergences are the unique family of f-divergences that can be estimated using our type of estimator based on waiting time random variable transformations. Take the vectorial case for example, where we construct kNN empirical density estimates for the probability densities ${\hat{p}}_{k},{\hat{q}}_{k}$; for an estimator that is based on these estimates to work for an f-divergence, we would require the f-divergence to be computable through an affinity term as an integration of the form $\int_{x}f\left(\frac{p(x)}{q(x)}\right)p\left(x\right)dx$ or $\int_{x}f\left(\frac{p(x)}{q(x)}\right)q\left(x\right)dx$ up to some constant terms, and we require that the affinity generating functions *f* satisfy a functional form that can be separated as either f(ab)=g(a)+h(b) or f(ab)=g(a)h(b) for some functions *g* and *h*. This restriction is made because, as we have seen for KL or α-divergence estimators, we rely on the independence property of the waiting time random variables, hence we can separate the empirical sums into three terms which converge separately and show the estimator to converge asymptotically up to additive or multiplicative bias constants. Let us examine these two types of restrictions on *f*.

For f to satisfy f(xy)=g(x)+h(y),∀x,y>0, we can see that f is equivalent to g and h in the sense that they differ by a constant. Differentiating the previous equation with respect to *x* and setting x=1 we would get:

$$f^{\prime }\left(y\right)=\frac{c}{y},$$

where $c=g^{\prime }\left(1\right)$ is a constant. The unique family of solutions to this condition is f(x)=clogx up to some additive constants. This obviously corresponds to KL divergence and reverse KL divergence when integrated against *P* and *Q*, respectively.

For the other case where f(xy)=g(x)h(y),∀x,y>0, let us consider differentiating both sides with respect to *x*; this gives:

$$f^{\prime }\left(xy\right)=g^{\prime }\left(x\right)\frac{h(y)}{y}.$$

Taking log on both sides and let $l=\log f^{\prime },m=\log g^{\prime }$:

$$l\left(xy\right)=m\left(x\right)+\log\frac{h(y)}{y}.$$

Now take the derivative with respect to *x* again and set x=1, we get:

$$l^{\prime }\left(y\right)=\frac{c}{y},$$

where $c=m^{\prime }\left(1\right)$ is a constant. The unique family of solutions satisfying the last condition is l(y)=clogy+C and hence $f\left(y\right)=ay^{b}$ is a general solution up to some additive constant. Without loss of generality, we can see that this corresponds uniquely to the affinity term of interest of the family of alpha divergences where $f\left(y\right)=y^{\alpha },\forall \alpha \in \left(0,1\right)$, and up to some constant terms.

Since KL and reverse KL divergence are limits of the α-divergences at two endpoints, we can conclude that the unique family of f-divergences that can be estimated based on the proposed estimators using waiting time random variables are the α-divergences. There is an interesting connection, pointed out by Amari [14], that states that α-divergence is the unique intersection between f-divergences and decomposable Bregman divergences on the space on positive measures. Notice that if restricted to the space of probability measures then the intersection reduces to only the KL divergences. Although the result does not directly connect to the uniqueness of α-divergences being estimable through our proposed methodologies, the proof technique that justifies the functional forms of the α-divergence being the unique f-divergence that allows a decomposition into the Bregman divergence dual functions up to some nonlinear coordinate transformation is very similar to what we carried out above and reaches the same conclusion—that the function *f* must take on a power function form that corresponds to an α-divergence and at the limit of α becomes logarithm functions that correspond to KL and reverse KL divergences.

## 7. Applications

The proposed estimator finds interesting applications in statistical estimation theory, clustering algorithms, visualization/embedding algorithms, and possibly online learning algorithms. We next describe a few such examples.

### 7.1. Bounding the Neyman–Pearson Region by Hellinger Affinity

We show that the Neyman–Pearson region contains one convex region determined by the Hellinger affinity, which is contained in another. These inclusion relations generalize the classical inequalities between total variation and Hellinger distance. Deploying our estimator for Hellinger affinity ${\hat{A}}_{S}\left(P,Q\right)$, we can approximately bound the Neyman–Pearson region.

Our results (see Appendix A for more details) show that, with two distributions p,q, and with s,t>0 (which can be chosen so that s+t=2 in standard case), the Neyman–Pearson region for type I (α(E)) and type II (β(E)) errors satisfies the following relation with the total variation distance for optimal choice of event $E^{★}$:

$$s\alpha \left(E^{★}\right)+t\beta \left(E^{★}\right)=\frac{t+s}{2}-\frac{1}{2}\int \left|sp-tq\right|d\mu ,$$

and can hence be bounded by the following inequalities where ρ(p,q) is the Hellinger affinity:

$$\frac{s+t}{2}-\sqrt{{\left(\frac{s+t}{2}\right)}^{2}-st\rho {\left(p,q\right)}^{2}}\leq s\alpha \left(E^{★}\right)+t\beta \left(E^{★}\right)\leq \sqrt{st}\rho \left(p,q\right).$$

Hence, by substituting our symmetric estimator for the Hellinger affinity term ${\hat{A}}_{S}\left(p,q\right)\approx \rho \left(p,q\right)$, we can approximately bound the Neyman–Pearson region given two samples from distributions *p* and *q*,

$$\frac{s+t}{2}-\sqrt{{\left(\frac{s+t}{2}\right)}^{2}-st{\hat{A}}_{S}{\left(p,q\right)}^{2}}⪅s\alpha \left(E^{★}\right)+t\beta \left(E^{★}\right)⪅\sqrt{st}{\hat{A}}_{S}\left(p,q\right).$$

If we are dealing with multivariate distributions, then the appropriate multivariate Hellinger affinity estimator from Section 4.3 can be used to approximately bound the Neyman–Pearson region. As a remark, we observe that there is no provable general relationship between Kullback–Leibler divergence or the rest of the α-divergences (besides Hellinger distance) with the Neyman–Pearson regions.

### 7.2. Estimating Eigenvalues of the Matrix Pencil for Inference in the Family of Concentric Gaussians

Consider two multivariate distributions from the concentric Gaussian family $P=N\left(0,C_{1}^{2}\right),Q=N\left(0,C_{2}^{2}\right)$, where $C_{1}^{2},C_{2}^{2}\in R^{d\times d}$. It can be shown that any meaningful statistical inference function on the two covariance matrices should satisfy $\phi \left(C_{1}^{2},C_{2}^{2}\right)=\phi \left(I,\Lambda \right)$, where Λ is the diagonal matrix with diagonal entries $\lambda_{1},\ldots ,\lambda_{d}$ being the eigenvalues of the matrix $C_{1}^{-1}C_{2}^{2}{\left(C_{1}^{-1}\right)}^{*}$; see Appendix C for more details.

Since Λ is diagonal and *I* is simply the identity matrix, we can write $\phi \left(C_{1}^{2},C_{2}^{2}\right)=h\left(\lambda_{1},\ldots ,\lambda_{d}\right)$. Hence, any inference we can make on the two concentric Gaussians will depend only on sufficient statistics, which are the eigenvalues $\lambda_{1},\ldots ,\lambda_{d}$. In the case of Hellinger affinity (and in general affinities for α-divergences), we can write it as $\phi \left(C_{1}^{2},C_{2}^{2}\right)=h\left(1,\lambda_{1}\right)\times \ldots \times h\left(1,\lambda_{d}\right)$, where $h(1,\lambda_{i}),\forall i=1,\ldots ,d$ is the affinity calculated based on two univariate Gaussian distributions N(0,1) and $N(0,\lambda_{i})$. For example, we have analytic formulas for the affinity term of the α-divergence family between such univariate Gaussian distributions:

$$h_{\alpha }\left(1,\lambda \right)=\sqrt{\frac{\lambda^{\alpha }}{\alpha \lambda +(1-\alpha )}}.$$

Then, we have, for the d-dimensional multivariate concentric Gaussians,

$$A^{\alpha }\left(P||Q\right)=\phi_{\alpha }\left(C_{1}^{2},C_{2}^{2}\right)=\sqrt{\frac{{\left(\prod_{i=1}^{d}\lambda_{i}\right)}^{\alpha }}{\prod_{i=1}^{d}\left(\alpha \lambda_{i}+1-\alpha \right)}}.$$

Now, given *d* distinct values of $\alpha_{1},\ldots ,\alpha_{d}$, the affinity values $A^{\alpha_{1}}\left(P||Q\right),\ldots ,A^{\alpha_{d}}\left(P||Q\right)$ can be used to determine the eigenvalues $\lambda_{1},\ldots ,\lambda_{d}$. Since our proposed estimator for vectorial α-affinities ${\hat{A}}_{k}^{\alpha }\left(P||Q\right)$ converges up to a multiplicative constant, we can use the estimated values for α-affinities corresponding to *d* different values of $\alpha =\alpha_{1},\ldots ,\alpha_{d}$ to estimate the eigenvalues $\lambda_{1},\ldots ,\lambda_{d}$ by solving a system of *d* equations. The estimated values ${\hat{\lambda }}_{1},\ldots ,{\hat{\lambda }}_{d}$ can be then used for any inference problems on these two probability distributions and they are sufficient for inference. This significantly reduces the noise in estimating the entire covariance matrices $C_{1}^{2},C_{2}^{2}$ when the data come from high dimensions where we could have an over-parametrization problem.

### 7.3. Stock Clustering and Visualization

We next describe a simple application to stock segmentation in a portfolio allocation setting. Consider N stocks with T historic dates. Let ${\left\{r_{i,t}\right\}}_{i\in [N],t\in [T]}$ denote the returns of each stock on each date. Let ${\left\{R_{i}\right\}}_{i\in [N]}$ denote the random variable standing for the returns of each stock, which is composed of data ${\left\{r_{i,t}\right\}}_{t\in [T}$. To cluster this universe of stocks into *K* distinct groups, we can first use the Hellinger distance estimator ${\hat{H}}_{S}\left(R_{i},R_{j}\right)$ for a pair of stocks ∀i≠j∈[N]. Since the estimator is symmetric, we would arrive at a symmetric distance matrix denoted by $D_{H}$. It is also possible to combine the Hellinger distance with a correlation distance metric through some transformations. After obtaining the distance matrix (or an affinity matrix by subtracting it from 1), we can deploy any desired clustering algorithm on it. The result would be *K* clusters of stocks that are grouped by similarity in the chosen distance sense. We can also add another step, which is to repair the distance matrix before clustering. There is the possibility that the distance matrix estimated using the proposed estimator does not exactly correspond to a metric, which means some groups of stocks may violate the triangle law in a metric. We can apply a simple sparse metric repair algorithm, see, for example, [15]. The resulting clustering can be helpful for portfolio allocation strategies since we can build sub-strategies inside each cluster and merge them together.

Another example using the same distance matrix constructed from sample data is in visualization algorithms such as FATE [16], which allow for the input of a precomputed distance/affinity matrix specifying the dataset. The visualization algorithm uses the input distance to compute embeddings in lower dimensions that preserve the local/global structures of the dataset and can be useful in many subsequent applications. Here, our estimator can also serve to compute the input distance matrix on sample data from N entities using the Hellinger distance or α-divergences as the distance metric. This could also be used in conjunction with a metric repair algorithm to adjust for the biases and errors in empirical estimators.

### 7.4. Other Applications

Lastly, we suspect that the proposed estimator can find interesting applications in UCB-type algorithms in multi-armed bandit frameworks, where the estimated pairwise Hellinger distances/α-divergences for sample distributions from different arms can be used to eliminate arms that fall outside of the confidence region balls around the top arms historically. We leave these open problems as future works.

## 8. Conclusions

We have proposed an estimator for the Hellinger affinity, and hence the squared Hellinger distance, between samples from two distributions based solely on the empirical CDF without the need to estimate the densities themselves. We have proven its almost-sure convergence to the true squared Hellinger distance and have constructed a symmetric version of this estimator. We showed the convergence behavior using several experiments where we observed that the symmetric estimator constructed from averaging the two one-sided estimators for the squared Hellinger distance turned out to be a favorable choice due to accuracy in general and smaller variances. We then extended the estimator to a family of α-divergences, where similar properties hold up to small modifications. For each choice of α, we also showed how to construct a symmetric version of the estimator. We also extended respective estimators to work with multivariate data in higher dimensions using k-nearest-neighbor-based estimators. Numerical examples are given to show the convergence of our proposed estimators. We conclude that the α-divergence family is the unique f-divergences that can be estimated consistently using the proposed methodologies. Our proposed estimators can be applied to approximately bounding the Neyman–Pearson region of a statistical test, among many other applications. 

## Appendix A. Shannon Entropy Estimator for 1D and Vectorial Data

Another simple extension of the methodologies in this work provides us with a convergent estimator for the Shannon entropy defined as:

$$H\left(P\right)=-\int_{x}p\left(x\right)\log p\left(x\right)dx$$

Here given 1D data samples ${\left\{x_{i}\right\}}_{i=1}^{n}$ from distribution P, we propose the following estimator:

$$\hat{H}\left(P\right)=-\frac{1}{n}\sum_{i=1}^{n}\log\delta P_{c}\left(x_{i}\right)$$

where $\delta P_{c}\left(x_{i}\right)$ are as defined in Section 4.1. It can be shown that:

$$\hat{H}\left(P\right)=-\frac{1}{n}\sum_{i=1}^{n}\log p\left(x_{i}\right)+\frac{1}{n}\sum_{i=1}^{n}\log\frac{\Delta P(x_{i})}{\Delta P_{c}\left(x_{i}\right)}=\frac{1}{n}\sum_{i=1}^{n}\log n\Delta P\left(x_{i}\right)-\frac{1}{n}\sum_{i=1}^{n}\log p\left(x_{i}\right)$$

This suggests that $\hat{H}\left(P\right)\to C+H\left(P\right)$ where $C=\int_{0}^{\infty }\log ze^{-z}dz\approx -0.5772$ is the Euler-Mascheroni constant. Hence we conclude that $\hat{H}\left(P\right)+0.5772$ converges almost surely to H(P), the true Shannon entropy of *P*.

Similarly, for vectorial data in d-dimensions, we define the estimator based on the k-nearest neighbor for a fixed *k*:

$${\hat{H}}_{k}\left(P\right)=-\frac{1}{n}\sum_{i=1}^{n}\log{\hat{p}}_{k}\left(x_{i}\right)$$

where ${\hat{p}}_{k}\left(x_{i}\right)$ is as defined in Section 4.3. By a similar argument, we show that:

$${\hat{H}}_{k}\left(P\right)=-\frac{1}{n}\sum_{i=1}^{n}\log p\left(x_{i}\right)+\frac{1}{n}\sum_{i=1}^{n}\log\frac{p(x_{i})}{{\hat{p}}_{k}\left(x_{i}\right)}\to H\left(P\right)+\frac{1}{(k-1)!}\int_{0}^{\infty }z^{k-1}\log ze^{-z}dz-\log k$$

Since the integral $\int_{0}^{\infty }z^{k-1}\log ze^{-z}dz$ evaluates to $\sum_{j=1}^{k-1}\frac{1}{j}+C$, we conclude that ${\hat{H}}_{k}\left(P\right)+\log k-\sum_{j=1}^{k-1}\frac{1}{j}+0.5772$ converges almost surely to H(P).

On a related note, a class of estimators of the Rényi and Tsallis entropies for multidimensional densities has been studied in [17], which is also based on computing the k-nearest neighbor distances from empirical samples.

## Appendix B. Hellinger Affinity and Neyman–Pearson Region

Following [18], we define the squared Hellinger distance and Hellinger affinity as:

$$H^{2}\left(p,q\right)=\frac{1}{2}\int {\left|\sqrt{p}-\sqrt{q}\right|}^{2}d\mu =1-\rho \left(p,q\right)$$

Then the Type I and Type II errors for any event *E* used as a test for distribution by *q*, are given by

$$\alpha \left(E\right)=\int_{E}pd\mu$$

$$\beta \left(E\right)=1-\int_{E}qd\mu$$

and we have the inequality for any non-negative s,t:

$$s\alpha \left(E\right)+t\beta \left(E\right)\geq \frac{t+s}{2}-\frac{1}{2}\int \left|sp-tq\right|d\mu$$

This is because $s\alpha \left(E\right)+t\beta \left(E\right)=t-\int_{E}\left(tq-sp\right)d\mu \geq t-\int_{E}\left|sp-tq\right|d\mu$, and $s\alpha \left(E\right)+t\beta \left(E\right)=s-\int_{E^{c}}\left(sp-tq\right)d\mu \geq s-\int_{E^{c}}\left|sp-tq\right|d\mu$, where $E^{c}$ is the complement of event *E*. Combining these results gives the aforementioned inequality, which can be seen as a generalization of the classic case when s=t=1, see Chapter 13 of [18]. For an optimal $E^{★}\left(s,t\right)$, an event for which this holds with equality (for example when $E^{★}\left(s,t\right)$ is the support of sp−tq<0), we have:

$$s\alpha \left(E^{★}\left(s,t\right)\right)+t\beta \left(E^{★}\left(s,t\right)\right)=\frac{t+s}{2}-\frac{1}{2}\int \left|sp-tq\right|d\mu$$

and $\left(\alpha \left(E^{★}\left(s,t\right)\right),\beta \left(E^{★}\left(s,t\right)\right)\right)$ is the point on the Neyman–Pearson boundary with supporting line $s\alpha \left(E\right)+t\beta \left(E\right)\geq s\alpha \left(E^{★}\left(s,t\right)\right)+t\beta \left(E^{★}\left(s,t\right)\right)$. Since the Neyman–Pearson region is convex, the family of f-divergences $\frac{1}{2}\int \left|sp-tq\right|d\mu$, where say s+t=2, obtains a complete description of the Neyman–Pearson region.

Additionally, we can relate the Neyman–Pearson region to the Hellinger distance by using the Hellinger affinity. It is convenient to compute

$$\frac{1}{2}\int {\left|\sqrt{sp}+\sqrt{tq}\right|}^{2}d\mu =\frac{s+t}{2}+\sqrt{st}\rho \left(p,q\right)$$

$$\frac{1}{2}\int {\left|\sqrt{sp}-\sqrt{tq}\right|}^{2}d\mu =\frac{s+t}{2}-\sqrt{st}\rho \left(p,q\right)$$

where the first term is from the conservation of probability. Then we use this to simplify the chain of inequalities:

$$\frac{1}{2}\int |\sqrt{sp}-\sqrt{tq}{|}^{2}d\mu \leq \frac{1}{2}\int |\sqrt{sp}-\sqrt{tq}|\left(\sqrt{sp}+\sqrt{tq}\right)d\mu =\frac{1}{2}\int \left|sp-tq\right|d\mu$$

and

$$\frac{1}{2}\int |sp-tq|d\mu =\frac{1}{2}\int \left|\sqrt{sp}-\sqrt{tq}\right|\left(\sqrt{sp}+\sqrt{tq}\right)d\mu$$

$$\leq (\frac{1}{2}\int |\sqrt{sp}-\sqrt{tq}{{|}^{2}d\mu )}^{\frac{1}{2}}{\left(\frac{1}{2}\int {\left(\sqrt{sp}+\sqrt{tq}\right)}^{2}d\mu \right)}^{\frac{1}{2}}$$

$$=\sqrt{{\left(\frac{s+t}{2}\right)}^{2}-st\rho {\left(p,q\right)}^{2}}$$

(where we used the Cauchy-Schwarz inequality) to the chain of inequalities:

$$\frac{s+t}{2}-\sqrt{st}\rho \left(p,q\right)\leq \frac{1}{2}\int \left|sp-tq\right|d\mu \leq \sqrt{{\left(\frac{s+t}{2}\right)}^{2}-st\rho {\left(p,q\right)}^{2}}$$

We obtain then for the Neyman–Pearson boundary the upper and lower bounds in terms of the Hellinger affinity:

$$\frac{s+t}{2}-\sqrt{{\left(\frac{s+t}{2}\right)}^{2}-st\rho {\left(p,q\right)}^{2}}\leq s\alpha \left(E^{★}\right)+t\beta \left(E^{★}\right)\leq \sqrt{st}\rho \left(p,q\right)$$

This bound has the gigantic advantage of also bounding the Neyman–Pearson region for joint distributions, such as the result of i.i.d. samples. There is no general relationship between Kullback–Leibler divergence and Neyman–Pearson regions since, say, for the Bernoulli family we can have a sequence of pairs $\left\{p_{k},q_{k}\right\}$ such that $H^{2}\left(p_{k},q_{k}\right)\to 0$ but $D_{KL}(p_{k}\left|\right|q_{k})+D_{KL}(q_{k}\left|\right|p_{k})\to \infty$.

## Appendix C. Sufficient Information Eigenvalues for Inference between Concentric Gaussians

Let $P=N\left(0,C_{1}^{2}\right),Q=N\left(0,C_{2}^{2}\right)$. For any meaningful statistical inference function on P,Q we require

$$\phi \left(C_{1}^{2},C_{2}^{2}\right)=\phi \left(XC_{1}^{2}X^{*},XC_{2}^{2}X^{*}\right)$$

where *X* is a coordinate transformation. Writing the QR decomposition for X=QR and let *R* be chosen so that $RC_{1}^{2}R^{*}=I$ (where *Q* is unitary), which is equivalent to the Cholesky factorization $C_{1}^{-2}=R^{*}R$. Define $C_{1}=R^{-1}$ then we have

$$\phi \left(C_{1}^{2},C_{2}^{2}\right)=\phi \left(I,Q\left(C_{1}^{-1}C_{2}^{2}{\left(C_{1}^{-1}\right)}^{*}\right)Q^{*}\right)$$

We choose *Q* so that it is the eigenvectors of the Hermitian matrix $C_{1}^{-1}C_{2}^{2}{\left(C_{1}^{-1}\right)}^{*}$. So we have the diagonal matrix $\Lambda =Q\left(C_{1}^{-1}C_{2}^{2}{\left(C_{1}^{-1}\right)}^{*}\right)Q^{*}$ where the diagonal elements are eigenvalues of $C_{1}^{-1}C_{2}^{2}{\left(C_{1}^{-1}\right)}^{*}$. Then we obtain,

$$\phi \left(C_{1}^{2},C_{2}^{2}\right)=\phi \left(I,\Lambda \right)$$
